# Platelet transfusion induces transfusion‐associated circulatory overload in rats with myocardial infarction

**DOI:** 10.1111/trf.18285

**Published:** 2025-05-19

**Authors:** Philippa G. Phelp, Britt Hurks, Chantal A. Polet, Joris J. T. H. Roelofs, Anita M. Tuip‐de Boer, Rick Kapur, Alexander P. J. Vlaar, Charissa E. van den Brom, Robert B. Klanderman

**Affiliations:** ^1^ Laboratory of Experimental Intensive Care and Anesthesiology Amsterdam University Medical Centers, University of Amsterdam Amsterdam The Netherlands; ^2^ Department of Intensive Care Medicine Amsterdam University Medical Centers, University of Amsterdam Amsterdam The Netherlands; ^3^ Department of Pathology Amsterdam University Medical Centers, University of Amsterdam Amsterdam The Netherlands; ^4^ Amsterdam Cardiovascular Sciences, Microcirculation Vrije Universiteit Amsterdam Amsterdam The Netherlands; ^5^ Sanquin Research, Department of Experimental Immunohematology, Amsterdam and Landsteiner Laboratory Amsterdam University Medical Centers, University of Amsterdam Amsterdam The Netherlands; ^6^ Department of Anesthesiology Amsterdam University Medical Centers, Vrije Universiteit Amsterdam Amsterdam The Netherlands; ^7^ Department of Anesthesiology Amsterdam University Medical Centers, University of Amsterdam Amsterdam The Netherlands

**Keywords:** acute lung injury, animal model, platelet transfusion, pulmonary edema, transfusion reaction, transfusion‐associated circulatory overload

## Abstract

**Background:**

Transfusion‐associated circulatory overload (TACO) accounts for 35% of transfusion‐related fatalities. Previous pre‐clinical studies explored plasma and red blood cell transfusion in TACO, but the effect of platelets remains unexplored. Platelet transfusions have the highest rate of adverse reactions and are associated with increased mortality in TACO patients. We aimed to determine whether platelet transfusion induces circulatory overload compared to crystalloids and whether it causes a more severe phenotype than plasma in a TACO rat model of myocardial infarction (MI).

**Methods:**

A validated TACO model in anemic Wistar rats with MI was used. Animals received platelets (*n* = 11), plasma (*n* = 10), or Ringer's lactate (*n* = 11). Pulmonary capillary pressure was assessed via left‐ventricular end‐diastolic pressure (LVEDP). The primary outcome was the change in LVEDP (ΔLVEDP) following transfusion. Secondary outcomes included pulmonary wet/dry weight ratio, oxygen tension or partial pressure of oxygen (PaO_2_)/fraction of inspired oxygen (FiO_2_) ratio, and circulating biomarkers.

**Results:**

ΔLVEDP following platelet transfusion (10.4 ± 4.6 mmHg) was significantly greater than Ringer's lactate (0.9 ± 1.4 mmHg; *p* < .001), but similar to plasma (13.0 ± 7.7 mmHg; *p* > .5). Pulmonary wet/dry weight ratios were comparable across groups (*p* > .5). At termination, PaO_2_/FiO_2_ ratio was significantly lower after platelet transfusion (372 ± 48) compared to Ringer's Lactate (447 ± 79; *p* < .05). N‐terminal prohormone of brain natriuretic peptide (NT‐proBNP) did not differ between groups at termination (*p* > .1). However, NT‐proBNP significantly increased from baseline (50 ± 24 pg/mL) to termination (177 ± 86 pg/mL) across all groups (*p* < .001).

**Discussion:**

Platelet transfusion induces circulatory overload in a TACO rat model with heart failure when compared to crystalloids, showing a trend toward reduced oxygenation compared to plasma transfusion. Further investigation is needed to determine the pathophysiological mechanisms.

**Abbreviations**
ΔLVEDP: difference in LVEDP pre‐transfusion compared to post‐transfusion
ANOVA: analysis of variance
ARRIVE: Animal Research: Reporting of In Vivo Experiments
Circ. vol: circulating volume
CO: cardiac output
CVP: central venous pressure
ECG: electrocardiogram
ELISA: enzyme‐linked immunosorbent assay
Hct: hematocrit
HR: heart rate
ICAM‐1: intercellular adhesion molecule 1
IL‐6: interleukin‐6
IQR: interquartile range
LAD: left‐anterior descending coronary artery
LVEDP: left‐ventricular end‐diastolic pressure
LVPmax: left‐ventricular maximum pressure
MAP: mean arterial pressure
MI: myocardial infarction
NT‐proBNP: N‐terminal pro‐brain natriuretic peptide
PaO2/FiO2 ratio: partial pressure of oxygen/fraction of inspired oxygen ratio
Pcap: pulmonary capillary pressure
PV: pressure‐volume
RBC: red blood cells
SD: standard deviation
SVR: systemic vascular resistance
TACO: transfusion‐associated circulatory overload
Term: termination
TNF‐α: tumor necrosis factor alpha
TRALI: transfusion‐related acute lung injury
Trx: transfusion

## 1 INTRODUCTION

Transfusion‐associated circulatory overload (TACO) is the leading cause of transfusion‐related morbidity and mortality,^1^, ^2^ responsible for 5% of all transfusion‐associated adverse reactions and 35% of transfusion‐related fatalities in the United States.^3^ TACO manifests as acute respiratory distress and/or pulmonary edema within 12 h post‐transfusion.^4^ The pathophysiology of TACO is hypothesized to involve a two‐hit mechanism.^2^, ^5^, ^6^ The first hit is volume incompliance due to the patient's pre‐existing clinical condition, such as cardiac or renal failure. The second hit is a blood transfusion, which overwhelms the circulation, increases pulmonary capillary pressure (Pcap), and drives transudate into the alveoli (hydrostatic pulmonary edema). However, the pathophysiology of TACO is more complex than simple volume overload, and the unknown underlying mechanisms hinder the development of targeted prevention and treatment.^7^

Previous animal models have provided valuable insights into TACO, utilizing a “two‐hit” hypothesis.^8^, ^9^, ^10^, ^11^ Transfusion of red blood cells (RBCs) alone does not significantly elevate Pcap (assessed as left‐ventricular end‐diastolic pressure [LVEDP]), whereas combining it with a “first hit” such as myocardial infarction (MI) induces a marked elevation.^8^ Additionally, both a faster speed and increased volume of transfusion are important in increasing TACO risk.^9^ Interestingly, volume reduced transfusion does not prevent TACO,^11^ strengthening the theory that the pathophysiology of TACO is more complex than pure volume overload. This research has focused on RBC and plasma transfusions.

However, the role of platelet transfusions in TACO remains unexplored. This is a significant gap, since platelet transfusions have the highest rate of adverse reactions among all blood components and are associated with an elevated risk of mortality in TACO patients.^1^, ^12^ In a retrospective observational study, the odds of surviving TACO were 1.97 times greater in patients with no platelet transfusions compared to those who received platelets.^12^ Platelet products are particularly volatile because they deteriorate rapidly and contain cellular products that increase the allogeneic exposure compared to plasma alone. Furthermore, platelets play a dual role in lung health: they help maintain vascular integrity and repair but can also contribute to lung injury.^13^ In transfusion‐related acute lung injury (TRALI), platelet transfusions can influence the development of respiratory distress by increasing pulmonary inflammation, coagulopathy, and endothelial injury.^14^, ^15^, ^16^, ^17^, ^18^, ^19^ Investigating the role of platelets in TACO is crucial, as platelets could exacerbate TACO through mechanisms such as inflammation, vascular permeability alterations, and endothelial interactions.

The primary aim of this study is to investigate whether platelet transfusion induces circulatory overload compared to crystalloids in a TACO rat model with heart failure. Additionally, our explorative secondary aim is to determine if platelet transfusion leads to a more severe TACO phenotype of acute lung injury or increased pulmonary edema, compared to plasma transfusion.

## 2 MATERIALS AND METHODS

### 2.1 Ethics and experimental set‐up

**FIGURE 1:**
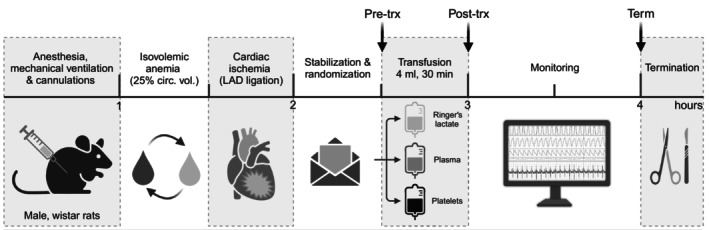
Experimental design of two‐hit transfusion‐associated circulatory overload model. Rats were anesthetized, mechanically ventilated, and cannulated for hemodynamic measurements. Anemia was induced by replacement of blood with an equal volume of colloid solution, reducing the hematocrit to ±30%. The first hit is cardiac ischemia, achieved by ligation of the left‐anterior descending coronary artery (LAD), rendering rats volume incompliant. Animals were randomized to receive Ringer's lactate infusion, plasma transfusion, or platelet transfusion. All groups received 4 units (4 mL) of the respective product over a period of 30 min. Animals were monitored up to 1 h after the end of transfusion. The primary points of measurement are indicated. Circ. vol., circulating volume; Pre‐trx, pre‐transfusion measurement; post‐trx, post‐transfusion measurement; term, termination measurement.

All experiments conducted received approval from the Institutional Animal Care and Use Committee of the University of Amsterdam, the Netherlands (license number: AVD11800202316816). Experiments were conducted following the EU Directive (2010/63EU) on the protection of vertebrate animals used for experimental and other scientific purposes and presented in accordance with ARRIVE (Animal Research: Reporting of In Vivo Experiments) guidelines.^20^ Male adult Wistar rats, weighing between 300 and 350 g (Envigo, The Netherlands), were utilized for this study. Animals were acclimatized for a minimum of 7 days in a specialized animal care facility before the start of the experiment and housed in cages containing two to five animals in a temperature‐controlled room (12/12 h light dark cycle, 20–23°C, 40%–60% humidity). During this period, rats were provided with standard rat chow and unrestricted access to water. The experimental overview is shown in Figure 1.

### 2.2 Anesthesia and analgesia

A validated, previously published, two‐hit TACO model was used.^8^, ^9^, ^10^ Animals were anesthetized by intra‐peritoneal injection of racemic ketamine (90 mg/kg; Alfasan, The Netherlands), dexmedetomidine (0.125 mg/kg; Orion Pharma, Finland) and atropine (0.025 mg/kg; Teva Nederland, The Netherlands) at a total volume of 1.5 mL/kg. Maintenance anesthesia was provided through a tail vein cannula with racemic ketamine (50 mg/kg/h) and dexmedetomidine (15 μg/kg/h) alongside Ringer's lactate (B.Braun, Germany) at a total volume of 4 mL/kg/h.

### 2.3 Ventilation

Animals received a tracheotomy and were mechanically ventilated (VentElite; Harvard Apparatus, USA). Our protocol was volume‐controlled ventilation, tidal volume of 6 mL/kg, a positive end‐expiratory pressure of 3 cmH_2_O and a fraction of 45% oxygen. Respiratory rate was 65 breaths/min and adjusted based on the arterial blood gas analyses (RapidPoint 500e; Siemens Healthineers, Germany). Body temperature was maintained at 37°C.

### 2.4 Surgical preparations

The right carotid artery was cannulated, and an ultra‐miniature rat pressure‐volume (PV) microcatheter (SPR‐838; Millar, USA) was inserted into the left ventricle. The right jugular vein was cannulated to monitor central venous pressure (CVP) and administer the transfusion. The left carotid artery was cannulated for monitoring mean arterial blood pressure (MAP) and blood sampling. A three‐lead electrocardiogram (ECG) was used to monitor heart rhythm, heart rate (HR), and to confirm MI. Hemodynamic data were continuously recorded using PowerLab 8/35 and LabChart 8 (ADInstruments, Australia).

### 2.5 Isovolemic anemia

Approximately 20% of estimated (based on body weight) circulating blood volume was withdrawn (at 16 mL/h) and replaced with an equal volume of colloid solution (16 mL/h, Volulyte 6%; Fresenius Kabi, Germany). Serial dilutions were performed until a hematocrit of 30% ± 2 was achieved.

### 2.6 First hit—heart failure

A MI was induced to impair cardiac performance. As previously described, a left‐anterior thoracotomy was performed and the left‐anterior descending coronary artery (LAD) was permanently ligated.^8^ Cardiac ischemia was confirmed by myocardial blanching and ST‐elevations on the ECG. A chest tube (20G) was placed below the incision and removed under negative pressure after thorax and skin closure (3‐0 Vicryl suture; Ethicon, USA), removing residual air while simultaneously performing a lung recruitment maneuver (tidal volume increased to 7.5 mL/kg for ±20 breaths). Norepinephrine infusion (2 μg/h; ±0.09 mcg/kg/min; Centrafarm, The Netherlands) was started at chest opening and titrated to a MAP of 55 mmHg. Animals were left to stabilize for 30 min to achieve stable hemodynamics post‐MI.

### 2.7 Randomization and second hit—transfusion

Animals were randomly assigned to one of three experimental groups using a sealed envelope, block randomization method: (1) platelet concentrate transfusion; (2) plasma transfusion; or (3) Ringer's lactate infusion, with all groups receiving equal volumes. Platelet transfusion was compared to Ringer's lactate to determine if TACO occurs with platelet transfusion. The plasma group was included to validate that our model was comparable to previous experiments and to determine if platelets caused a more severe phenotype than plasma. Randomization occurred after sufficient stabilization post‐isovolemic anemia and MI. Researchers were not blinded due to the preparation required for each transfusion product. After randomization, no changes were allowed to ventilator pressures, fluids, norepinephrine, and anesthetic infusion rates. A fixed volume of 4 mL (human equivalent of 4 units) was transfused via the internal jugular line over 30 min using a volumetric pump. Hemodynamic parameters were continuously recorded until 1 h post‐transfusion, after which animals were euthanized by exsanguination.

### 2.8 Hemodynamic measurements

Hemodynamic measurements were taken at fixed intervals, with beat‐to‐beat data averaged over 1 min: pre‐transfusion, midway through transfusion, immediately post‐transfusion, and at 15–30 and 60 min post‐transfusion (Figure 1). Parameters recorded included HR, MAP, CVP, left ventricular pressure (including LVEDP) and volume. Cardiac output and systemic vascular resistance (SVR) were calculated from the PV data. The PV catheter was calibrated for change in blood conductivity using volume cuvettes, and myocardial parallel conductance was measured via hypertonic saline bolus injections (30% NaCl, 20 μL), following best‐practice protocols.^21^

### 2.9 Preparation of transfusion products

Plasma and platelet products were prepared from whole blood harvested from exsanguinated donor animals. Donor animals were anesthetized with 5% isoflurane (30% O_2_), and blood was aspirated through a closed‐chest left‐ventricular puncture. Blood products were processed per Dutch Blood Banking standards and as previously described.^18^ Whole blood was collected in citrate–phosphate‐dextrose solution (1:8; Sigma‐Aldrich, USA) and rested for 4 h at room temperature. After centrifugation (15 min, 2110 g, 20°C), the plasma and buffy coat were isolated. The buffy coat was centrifuged again (10 min, 288 g, 20°C) to remove residual RBCs and leukocytes. Platelet concentrate from all five donors was pooled, diluted with plasma to a platelet count of ±1000 × 10^9^/L, and stored for a maximum of 3 days at ±22°C in horizontally shaking culture flasks (Greiner bio‐one, Germany) under 5% CO_2_. The remaining plasma was centrifuged again (15 min, 2110 g, 20°C) to obtain platelet‐poor plasma for the plasma transfusion group. Filtration of plasma was not performed to limit loss of product volume in the filter, necessitating more donor animals. Plasma products were stored at −20°C for up to 1 month, thawed at 37°C, and filtered (0.2 μm) before transfusion. Platelet quality was assessed by pH, lactate (RapidPoint 500e), and platelet activation by quantifying CD62 positive platelets using flow cytometry (PE anti‐rat CD62P, Cat# 148306 and Allophycocyanin anti‐rat CD61, Cat# 104315, BioLegend, USA).

### 2.10 Pulmonary edema

The right lung was harvested, and the right middle lower lobe and the right lower lobe were weighed immediately (wet weight), then dried at 37°C for 7 days before weighing again (dry weight). The lung wet‐to‐dry weight ratio was calculated to quantify pulmonary edema.

### 2.11 Pulmonary histology

The right upper lobe was fixed in 4% formalin and then processed into hematoxylin and eosin‐stained slides for histopathological analysis by an experienced pathologist. The extent of perivascular and alveolar edema was ranked on a 0‐ to 3‐point scale based on the guidelines for scoring acute lung injury in rodents.^22^, ^23^ Histopathological scoring was performed based on the severity and distribution of edema within the lung tissue.^22^, ^23^

### 2.12 Cardiac infarct

The heart was harvested with the LAD suture in place, and the infarct size was quantified to ensure equal consistency across groups. The aorta was cannulated and coronary circulation perfused with: 5 mL heparinized saline (1.0 IE/mL; LEO Pharma, Denmark) to prevent clots, then with 1 mL 2% Evans Blue dye (Sigma‐Aldrich, USA) to identify the area at risk of ischemia, and then with saline to flush out excess dye. Hearts were frozen at −20°C for up to 2 weeks. Frozen hearts were cut into 2 mm transverse slices and counterstained with triphenyltetrazolium chloride (7.5 mg/mL; Sigma‐Aldrich, USA) at 37°C for 20 min, then fixed in 10% formalin (Merck, USA) for 2 h. Slices were imaged (CANON EOS 60D) on a green background to enhance contrast. Infarct size was quantified using ImageJ (1.49v/Java 1.8.0_45 [64‐bit]).

### 2.13 Plasma analysis

Blood samples from baseline and termination were centrifuged (10 min, 2000 g, 4°C) and plasma supernatant was stored at −70°C. Interleukin‐6 (IL‐6), tumor necrosis factor alpha (TNF‐α), and intercellular adhesion molecule 1 (ICAM‐1) (markers of inflammation and endothelial activation) were measured using a custom rat pre‐mixed multi‐analyte Luminex assay (R&D Systems, USA). N‐terminal prohormone of brain natriuretic peptide (NT‐proBNP; Cat# BI‐1204R, Biomedica, Austria), angiopoietin‐2 (Cat# SEA009Ra, Cloud‐Clone Corp., USA) and syndecan‐1 (Cat# MBS2703971, MyBioSource, USA) levels, markers for circulatory overload and endothelial and glycocalyx damage, were quantified using ELISA (enzyme‐linked immunosorbent assay) following manufacturers' guidelines.

### 2.14 Sample size

The primary outcome was the change in LVEDP (∆LVEDP) from pre‐ to post‐transfusion across groups. Based on pilot data and a previously published TACO MI model,^10^ a sample size of *n* = 11 per group was calculated using nQuery (v8.5.1, Dotmatics, USA) to compare platelets (+7.8 mmHg, SD = 5.6) with Ringer's lactate (+2.0 mmHg, SD = 4.0). This calculation assumed a standard deviation of 4.5 mmHg, *α* = .05, and 80% power. This study is not powered to detect differences between plasma and platelet transfusion, as this is an explorative secondary hypothesis.

### 2.15 Statistical analysis

Animals with MAP <45 mmHg or that died before transfusion were excluded and replaced. Statistical analysis was performed using GraphPad Prism (v10.2.0, GraphPad Software, USA). A Shapiro–Wilk test was applied to all data to assess normality, supplemented by a visual assessment of Quantile‐Quantile plots. Given the small sample size, normality testing is challenging; thus, we aimed for consistency by using a single test for all data. Experiments were reviewed for reliability, and exclusions were made only for clear reasons (e.g., experimental error or failure to meet pre‐transfusion criteria). Outliers were not removed without specific justification. ΔLVEDP and secondary outcomes were compared using one‐way ANOVA with Bonferroni's multiple comparisons test or Kruskal–Wallis with Dunn's multiple comparisons test, as appropriate. Change over time were analyzed with two‐way repeated‐measures ANOVA (analysis of variance). Significance was set at *p* < .05. Parametric data are expressed as mean ± standard deviation (SD), and non‐parametric data as median with interquartile range (IQR).

## 3 RESULTS

Prior to randomization, two animals died from ventilator malfunction and blood loss due to cannulations. Four animals were excluded due to hemodynamic collapse (MAP <45 mmHg, *n* = 3) after MI or failed PV‐catheter placement (*n* = 1). One animal was excluded post‐randomization due to a solitary kidney, which could affect fluid dynamics.

Pre‐transfusion baseline characteristics were comparable between all groups (Table 1). Isovolemic anemia was achieved, and MI was confirmed in all animals, with the resulting size of the MI comparable across all groups.

**TABLE 1:** Pre‐transfusion characteristics.

Characteristic	Ringer's (*n* = 11)	Plasma (*n* = 10)	Platelets (*n* = 11)	*p*‐Value
Weight (g)	330 ± 12	327 ± 10	328 ± 12	.79
Age (days)	71 [70–72]	74 [72–77]	71 [71–77]	.20
Infarct size (%)	32 ± 9	33 ± 9	29 ± 12	.64
LVEDP (mmHg)	8.5 [7.1–10.6]	9.3 [7.4–11.5]	7.7 [6.6–9.7]	.40
Heart rate (min^−1^)	264 [258–286]	267 [253–289]	269 [256–317]	.72
MAP (mmHg)	61 [55–66]	55 [53–57]	57 [53–73]	.14
LVP_max_ (mmHg)	82 ± 9	87 ± 7	86 ± 15	.54
Stroke volume (mL)	48 ± 14	64 ± 23	52 ± 13	.10
CO (mL/min)	13.1 ± 4.0	17.5 ± 6.7	14.6 ± 3.4	.12
SVR (dyn s/cm^5^)	398 ± 162	273 ± 92	339 ± 81	.07
CVP (mmHg)	2.0 ± 0.7	1.7 ± 0.6	2.2 ± 0.9	.28
Fluid input (mL/kg)	18.8 ± 2.9	20.5 ± 3.6	20.4 ± 2.6	.35
Vent duration (min)	165 ± 14	177 ± 15	172 ± 20	.28
pH	7.36 ± 0.02	7.35 ± 0.03	7.37 ± 0.02	.40
pCO_2_ (mmHg)	35.4 ± 4.0	35.3 ± 5.9	34.5 ± 3.0	.86
pO_2_ (mmHg)	185 ± 40	189 ± 36	191 ± 33	.92
Hct (%)	38.9 ± 1.2	38.9 ± 1.6	39.2 ± 1.3	.86
Lactate (mmol/L)	2.9 ± 0.8	3.2 ± 0.7	3.2 ± 1.0	.67

*Note*: Characteristics at randomization, prior to transfusion. Data are presented as mean ± SD or median [IQR].

Abbreviations: CO, cardiac output; CVP, central venous pressure; Hct, hematocrit; LVEDP, left‐ventricular end‐diastolic pressure; LVP_max_, left‐ventricular maximum pressure; MAP, mean arterial pressure; SVR, systemic vascular resistance.

### 3.1 LVEDP increased significantly after transfusion of both platelets and plasma

**FIGURE 2:**
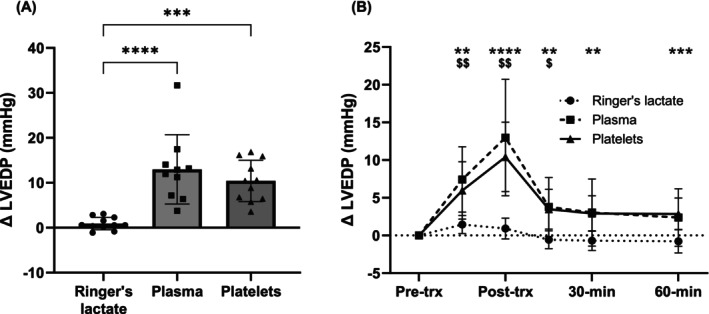
The effect of transfusion on left‐ventricular end‐diastolic pressure (LVEDP). (A) Change in LVEDP from pre‐transfusion to post‐transfusion following infusion of Ringer's lactate or transfusion of plasma or platelets. Each dot represents an individual rat. (B) The change in LVEDP over the course of the experiment, where the ΔLVEDP is the change in LVEDP at each time point compared to pre‐transfusion LVEDP. ^$^Plasma compared to Ringer's lactate. Data are presented as mean ± SD. ^$^ *p* < .05; **/^$$^ *p* < .01; ***p < .001; ****p < .0001. trx, transfusion.

Platelet transfusion (10.4 ± 4.6 mmHg) resulted in a significantly higher ΔLVEDP compared to Ringer's lactate (0.9 ± 1.4 mmHg, *p* = .0005, Figure 2A). The increase in LVEDP following platelet transfusion did not differ significantly from the increase in LVEDP following plasma transfusion (13.0 ± 7.7 mmHg, *p* = .80). At 60‐min post‐transfusion, the ΔLVEDP (termination LVEDP—pre‐transfusion LVEDP) was still greater in the platelet group (*p* = .0006) compared to Ringer's lactate, but not in the plasma group (*p* = .1) (Figure 2B).

### 3.2 Platelet and plasma transfusion resulted in TACO‐related hemodynamic changes

**FIGURE 3:**
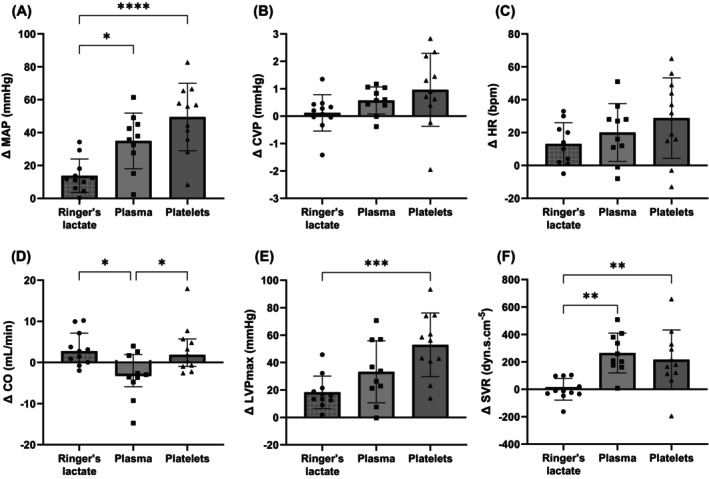
Change in hemodynamics following transfusion. (A) ΔMAP, (B) ΔCVP, (C) ΔHR, (D) ΔCO, (E) ΔLVPmax, (F) ΔSVR. Each dot represents an individual rat. Cardiac output (CO) data is presented as median [IQR], and all other data is presented as mean ± SD. *p < .05; **p < .01; ***p < .001; ****p < .0001. CVP, central venous pressure; HR, heart rate; LVPmax, left‐ventricular maximum pressure; MAP, mean arterial pressure; SVR, systemic vascular resistance.

Platelet transfusion resulted in a significantly greater ΔMAP (+49.5 ± 20.5 mmHg) compared to Ringer's lactate (+13.8 ± 10.2 mmHg, *p* < .0001) (Figure 3). The ΔMAP did not differ between platelet and plasma transfusion (+34.9 ± 16.9 mmHg, *p* = .16). ΔCVP and ΔHR did not differ between groups (*p* = .12 and *p* = .19, respectively). ΔCardiac output was significantly lower following plasma transfusion (−3.3 mL/min, IQR: −5.9 to 1.9) when compared to both Ringer's lactate infusion (+2.7 mL/min, IQR: 0.1–7.1, *p* = .017) and platelet transfusion (+1.9 mL/min, IQR: −1.0 to 5.7, *p* = .039). Platelet transfusion (33 ± 23 mmHg) resulted in a significantly greater increase in LVP_max_ (left‐ventricular maximum pressure) when compared to Ringer's lactate infusion (18 ± 12 mmHg, *p* = .0009). Platelet transfusion (216 ± 217 dyn s/cm^5^) resulted in a significant larger ΔSVR compared to Ringer's lactate infusion (0 ± 79 dyn s/cm^5^, *p* = .01). The ΔSVR did not differ between platelets and plasma (264 ± 145 dyn s/cm^5^, *p* > .99).

### 3.3 Pulmonary outcomes

**FIGURE 4:**
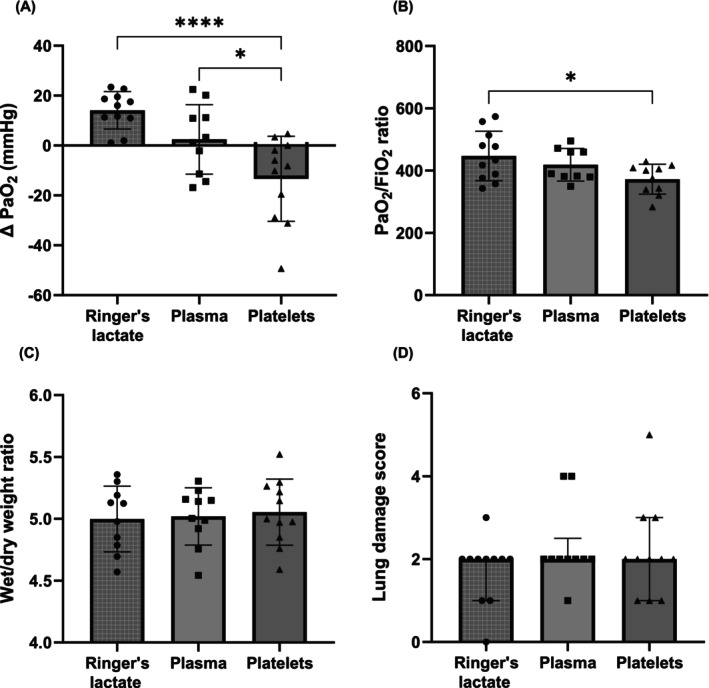
Pulmonary outcomes. (A) Change in oxygen tension. (B) Termination oxygen tension or partial pressure of oxygen (PaO_2_)/fraction of inspired oxygen (FiO_2_) ratio. (C) Pulmonary wet/dry weight ratio. (D) Pathology score. Each dot represents an individual rat. Pathology scoring is presented as median [IQR], and all other data are presented as mean ± SD. *p < .05; ****p < .0001.

Platelet transfusion (−13 ± 17 mmHg) caused a significant decrease in oxygen tension in comparison to both Ringer's lactate (14 ± 8 mmHg, *p* < .0001) and plasma (2 ± 14 mmHg, *p* = .02) (Figure 4A). In addition, oxygen tension or partial pressure of oxygen (PaO_2_)/fraction of inspired oxygen (FiO_2_) ratio (measure of oxygen efficiency) at termination was significantly lower in animals that received platelets (372 ± 48) compared to Ringer's lactate (447 ± 79, *p* = .033), although there was no difference when compared to plasma (419 ± 52, *p* = .35) (Figure 4B). There was no difference in pulmonary wet/dry weight ratio (Figure 4C) nor histopathological lung injury (Figure 4D) between all groups.

### 3.4 Markers of circulatory overload and inflammation are increased in all intervention groups

**FIGURE 5:**
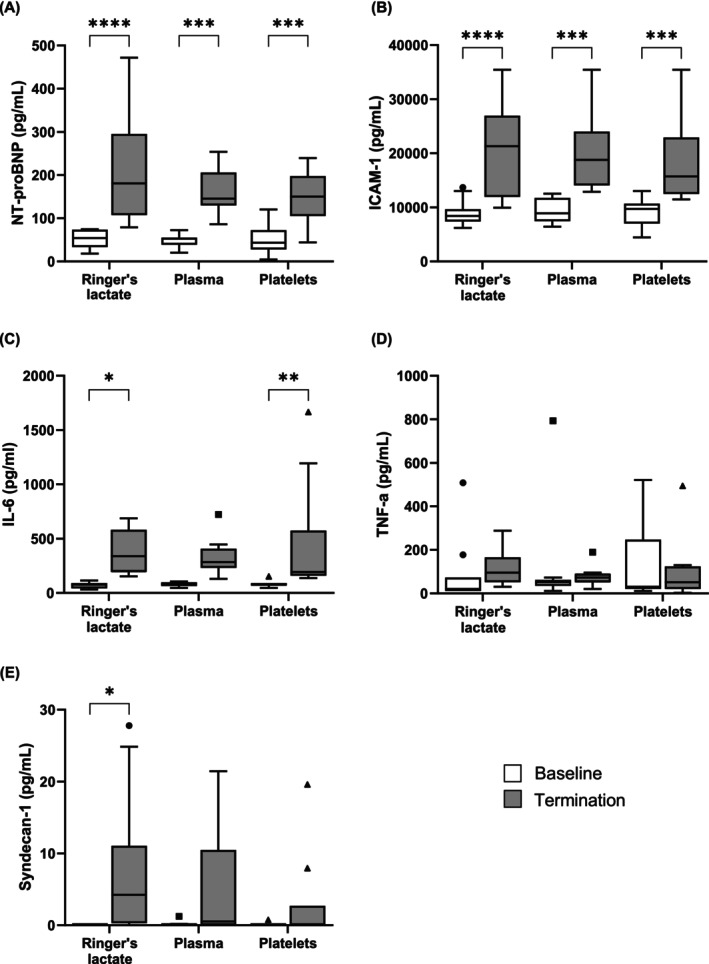
Plasma biomarkers at baseline and termination. (A) N‐terminal prohormone of brain natriuretic peptide (NT‐proBNP) levels, (B) intercellular adhesion molecule 1 (ICAM‐1) levels, (C) interleukin‐6 (IL‐6) levels, (D) tumor necrosis factor alpha (TNF‐α) levels, and (E) Syndecan‐1 levels. Each dot represents an individual rat. Data are presented in a Tukey boxplot. *p < .05; **p < .01; ***p < .001; ****p < .0001.

There was a significant increase in NT‐proBNP and ICAM‐1 from baseline to termination in all groups (Figure 5A,B). IL‐6 significantly increased following Ringer's lactate infusion and platelet transfusion, however, although there was an increase trend following plasma transfusion, this was not significant (Figure 5C). TNF‐α levels did not change from baseline to termination across all groups (Figure 5D). Syndecan‐1 significantly increased following Ringer's lactate infusion (Figure 5E). Overall, there was no significant difference in biomarkers at baseline or termination between the three intervention groups.

## 4 DISCUSSION

TACO after platelet transfusion has an increased mortality; however, no studies to date have specifically investigated why platelets may exacerbate mortality in TACO.^12^ This study evaluated the impact of platelet transfusion on TACO development in a validated TACO rat model. The main findings of this study are: (1) platelet transfusion significantly increased LVEDP compared to Ringer's lactate; (2) the increase in LVEDP is comparable between platelet and plasma transfusion; (3) both platelet and plasma transfusions showed TACO‐related hemodynamic changes, including increased blood pressure and heart rate; (4) platelet transfusion impaired oxygenation compared to Ringer's lactate and plasma; and (5) despite increased LVEDP, there were no signs of pulmonary edema. Additionally, circulating markers for circulatory overload, inflammation, endothelial activation, and endothelial damage increased across all groups at termination. Therefore, platelet transfusion induces circulatory overload in a TACO rat model with heart failure when compared to crystalloids, showing a trend toward reduced oxygenation compared to plasma transfusion.

We showed that platelet transfusion increases LVEDP in rats with myocardial dysfunction. Moreover, this increase is clinically relevant^24^ and comparable to previous research assessing RBC and plasma transfusion products.^8^, ^9^, ^10^, ^11^ Despite increased LVEDP, there was no corresponding rise in pulmonary edema, although edema was observed in all three transfusion groups compared to healthy control animals (Supporting Information S1). This could be attributed to the short follow‐up time, as TACO can develop within 12 h post‐transfusion. The absence of acute lung injury suggests that a priming inflammatory event, which has been linked to the pathophysiology of TACO, may be necessary for severe pulmonary manifestations. Clinical observations of elevated cytokines, such as IL‐6,^25^ and fever in TACO,^26^ suggest that pre‐existing inflammation may amplify lung injury. The increase in LVEDP did not result in a greater increase in NT‐proBNP, a biomarker supportive of TACO development. NT‐proBNP, however, can also increase after MI^27^ and it may require more time to increase significantly. The difficulties in employing NT‐proBNP in our study as well as various patient populations^28^ underscore the complexity of TACO pathophysiology and the likely interplay of additional inflammatory and clinical factors.

We hypothesized that platelet transfusion will not only induce circulatory overload but also lead to a more severe TACO phenotype than plasma transfusion. Both transfusions resulted in a comparable increase in LVEDP and TACO‐related hemodynamic changes, as well as similar levels of pulmonary edema, histological lung injury, and NT‐proBNP levels. However, platelet transfusion showed a trend toward greater oxygenation impairment. Although the mechanisms behind this cannot be determined from our study, platelets are increasingly recognized for their non‐hemostatic immune functions.^29^, ^30^ Activated platelets are known to induce neutrophil extracellular traps, which may increase endothelial damage and permeability.^31^ The higher resulting platelet count in the platelet group could have contributed to greater endothelial damage, leading to reduced oxygen efficiency. However, given the sample size and the presence of outliers, it is unclear whether this difference reflects a true biological effect or statistical variability. A larger study, powered to detect platelet‐plasma differences, is needed to determine whether platelet transfusion has a distinct impact on oxygenation impairment and the potential to cause a more severe TACO phenotype.

It is important to note that the platelets used in this study were prepared in plasma rather than platelet additive solution (PAS). This is comparable to clinical data which showed an increased mortality in TACO patients receiving platelets prepared in plasma.^12^ PAS‐platelets have a lower colloid osmotic pressure than fresh frozen plasma.^32^ Therefore, it is possible that PAS‐platelets would lead to a smaller LVEDP increase. Future studies should explore if PAS‐prepared platelets reduce pulmonary pressure and TACO risk compared to plasma‐prepared platelets.

This study has several strengths, including the use of a validated TACO rat model with MI, which simplifies TACO development. Precise hemodynamic measurements allowed for a detailed assessment of the cardiovascular impact of different transfusion products, focusing on their effects on LVEDP. While LVEDP is an approximation of Pcap, it remains the most robust and practical measurement available in this setting. Direct measurement of Pcap is technically challenging due to ventilatory and cardiac oscillations. Pulmonary capillary wedge pressure, as an alternative, has its own limitations, including greater susceptibility to variations in pH, oxygenation, and ventilatory pressures. By analyzing changes in LVEDP rather than absolute values, we account for any variation in LVEDP due to MI. A clinically relevant rat platelet product was used, stored for a maximum of 3 days with minimal handling and activation, representing a “good” platelet product. This allowed us to assess TACO development in the absence of factors present in aged platelets, such as lipids and microparticles, shown to contribute to the development of TRALI.^19^ The impact of prolonged storage of platelet products on the development of TACO is an interesting topic for further research.

This study has several limitations. Firstly, the short follow‐up time post‐transfusion might have been too short for additional edema due to transfusion to develop. Secondly, our study was not specifically powered to determine the difference between plasma and platelet transfusions. This limits the conclusions that can be drawn from this study.

## 5 CONCLUSION

In conclusion, this study demonstrates that platelet transfusion in a TACO rat model with myocardial impairment significantly increases Pcap (LVEDP) and induces oxygenation impairment compared to Ringer's lactate. However, whether platelet transfusion leads to a more severe TACO phenotype compared to plasma transfusion remains unclear. Both platelet and plasma transfusions resulted in comparable increases in LVEDP, and while platelet transfusion was associated with reduced oxygenation, it cannot be determined from this study whether this effect differs from plasma transfusion. Clinically, this highlights the importance of cautious use of both plasma and platelet transfusions, particularly in high‐risk patients. Further research is essential to clarify the underlying mechanisms driving TACO and to identify transfusion product risk factors, paving the way for improved prevention and management strategies.

## AUTHOR CONTRIBUTIONS

PGP, RBK, CAP, CEvdB, and APJV designed the experiment. PGP, BH, CAP, and AMT‐dB performed the research. RK provided expertise and advice. JJTHR assisted in reviewing the results. CEvdB, RBK, and APJV supervised the study. PGP, CEvdB, RBK, and APJV wrote the manuscript. All authors critically edited the manuscript.

## FUNDING INFORMATION

This research is supported by a Landsteiner Foundation for Blood Transfusion Research (LSBR) fellowship grant to Alexander P. J. Vlaar, number 1931F and a personal grant to Alexander P. J. Vlaar of NWO (Dutch: Nederlandse Organisatie voor Wetenschappelijk Onderzoek), VIDI grant number: 09150172010047.

## CONFLICT OF INTEREST STATEMENT

The authors have disclosed no conflicts of interest.

## Supporting information


**Data S1.** Supporting Information.
