# Supplementary material for: Platelet transfusion induces transfusion‐associated circulatory overload in rats with myocardial infarction
Source: Transfusion. 2025 May 19;65(7):1251–63. doi: 10.1111/trf.18285 (PMC12227162; doi:10.1111/trf.18285)
Supplement: Supplementary file 1 — Data S1. Supporting Information. [file TRF-65-1251-s001.docx]

**Supplementary material**

**
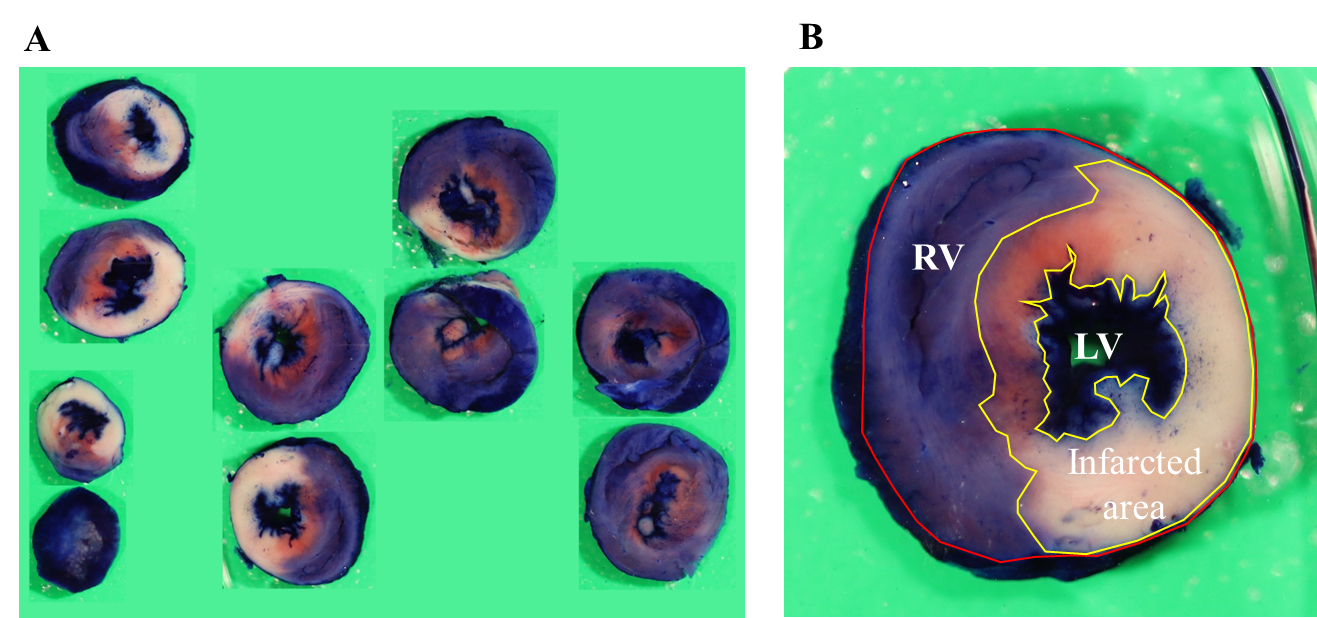
**

**Figure S1:** **A.** Representative image of triphenyltetrazolium chloride stained heart slices, with white area representing the infarcted area. **B.** Close up image of representative heart slice indicating how the infarcted area was measured. RV: right ventricle; LV: left ventricle.


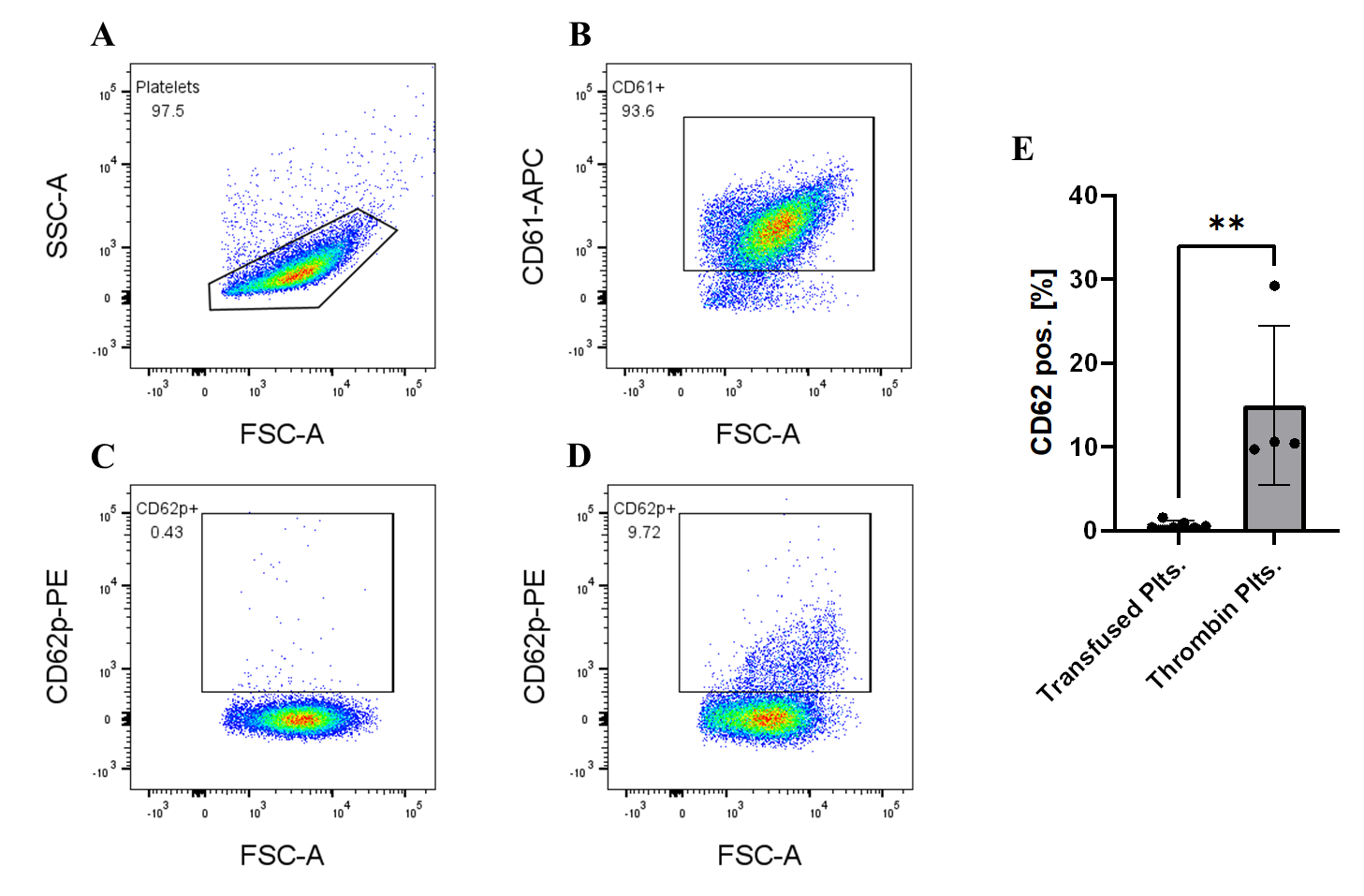


**Figure S2:** Platelet aliquots were taken from the prepared platelet products and analyzed for activation using CD62p on the cell surface measured with flow cytometry. **A.** Platelets were roughly gated from the platelet product. **B.** Platelet measurement was confirmed, and cell debris was excluded using CD61 as platelet marker. **C.** Platelet activation was assessed by measuring CD62p expression prior to transfusion. **D.** Platelets were activated using thrombin to confirm the potential for platelet activation. **E.** Percentages of activated platelets in unstimulated samples were compared to thrombin stimulated platelets.

**Figure S3:** Pulmonary wet/dry weight ratio of Ringer’s lactate, plasma and platelet transfusion compared to healthy control animals. These are animals that have been exsanguinated in order to obtain blood for transfusions products. After exsanguination, lungs were removed and weighed in the same manner as experimental animals. Data are presented as mean ± SD. **: p < 0.01; ***: p < 0.001.

| **Characteristics** | **Ringers (n=11)** | **Plasma (n=10)** | **Platelets (n=11)** | **p-value** |
| --- | --- | --- | --- | --- |
| *LVEDP (mmHg)* | 7.2 [6.8 - 11.3] | 12.1 [10.0 - 14.6] | 10.0 [8.6 - 15.3] | 0.06 |
| *Heart rate (min^-1^)* | 298 ± 37 | 298 ± 31 | 320 ± 35 | 0.26 |
| *MAP (mmHg)* | 69 ± 13 | 77 ± 19 | 87 ± 21 | 0.09 |
| *LVP_max_ (mmHg)* | 98 ± 14 | 109 ± 20 | 116 ± 23 | 0.11 |
| *Stroke Volume (mL)* | 54 ± 17 | 54 ± 24 | 59 ± 18 | 0.80 |
| *Cardiac output (mL/min)* | 16.4 ± 6.1 | 16.5 ± 8.2 | 18.7 ± 5.1 | 0.65 |
| *SVR (dyn*s/cm^5^)* | 300 [232 – 532] | 338 [292 – 653] | 407 [272 – 477] | 0.67 |
| *CVP (mmHg)* | 2.1 ± 0.5 | 2.1 ± 0.8 | 2.4 ± 1.0 | 0.54 |
| *Fluid input (mL/kg)* | 40.5 ± 3.0 | 39.6 ± 4.0 | 40.5 ± 3.1 | 0.78 |
| *Urine (mL)* | 6.0 [4.0 - 8.0] | 7.3 [5.8 - 11.8] | 10.5 [6.0 - 11.3] | 0.36 |
| *pH* | 7.33 ± 0.03 | 7.37 ± 0.03 | 7.37 ± 0.03 | 0.01 |
| *pCO_2_* | 34.5 ± 3.6 | 37.4 ± 6.0 | 39.7 ± 3.9 | 0.04 |
| *pO_2_* | 200 ± 44 | 193 ± 35 | 171 ± 29 | 0.21 |
| *P/F ratio* | 447 ± 79 | 419 ± 52 | 372 ± 48 | 0.04 |
| *Hct (%)* | 38 [36 – 40] | 35 [31 – 36] | 34 [34 – 35] | 0.00 |
| *Lactate* | 3.1 ± 1.1 | 3.1 ± 1.5 | 2.5 ± 0.8 | 0.42 |

**Table S1:** Termination characteristics. Data are presented as mean ± SD or median [IQR]. Abbreviations: LVEDP: left-ventricular end diastolic pressure; MAP: mean arterial pressure; LVP_max_: left-ventricular maximum pressure; SVR: systemic vascular resistance; CVP: central venous pressure; Hct: hematocrit.
